# Socio-economic impact of Rift Valley fever to pastoralists and agro pastoralists in Arusha, Manyara and Morogoro regions in Tanzania

**DOI:** 10.1186/2193-1801-2-549

**Published:** 2013-10-18

**Authors:** Augustino A Chengula, Robinson H Mdegela, Christopher J Kasanga

**Affiliations:** Department of Veterinary Microbiology and Parasitology, Faculty of Veterinary Medicine, Sokoine University of Agriculture, P O Box 3019, Morogoro, Tanzania; Department of Veterinary Medicine and Public Health, Faculty of Veterinary Medicine, Sokoine University of Agriculture, P O Box 3019, Morogoro, Tanzania

**Keywords:** Rift Valley fever, Socio-economic losses, Awareness, Pastoralists, Tanzania

## Abstract

Rift Valley fever (RVF) is a viral notifiable zoonotic disease primarily of domestic ruminants that causes significant socio-economic impacts. Using the 2006–07 outbreak cases, this study aimed to establish the socio-economic impact of RVF and assessing knowledge, attitude and practice of livestock keepers towards controlling RVF in selected areas of Tanzania. Data were collected in Arusha, Manyara and Morogoro regions using questionnaires, focus group discussions and in-depth interviews with key informants. Results indicate that there was little knowledge on disease (all clinical signs scored <50%) and the difference between the three regions was statistically significant (P = 0.00459). Socio-economic impacts of RVF shown by this study included; animal and human deaths, disruption of livestock market chains, inability of pastoralists to achieve their daily demands, inability to obtain protein leading to malnutrition and monetary loss at individual and national level during control of the disease. These findings have demonstrated low knowledge of the community on RVF, thus, more education and engagement is needed in order to develop more effective and efficient control strategies.

## Background

Rift Valley fever (RVF) is an acute vector-borne viral zoonotic disease affecting domestic animals and humans (Davies and Martin 2006). The disease is caused by the Rift Valley fever virus (RVFV), a member of the genus Phlebovirus of the family Bunyaviridae (Elliott 1997; Elliott 1990). The transmission of RVFV in domestic animals is either through bites from different species of infected mosquitoes, mainly the *Aedes* and *Culex* genera or by direct contact with infected animal tissues, bodily fluids and fomites, particularly if associated with abortions (Davies and Martin 2006; Soti et al. 2013). Transmission of the virus to humans is thought to occur by arthropod vectors, aerosols of blood or amniotic fluid, or other direct contact with infected animals (Woods et al. 2002). The relative importance of each mode of transmission varies according to the stage of the epizootic: in the first stage, the bites of infected mosquitoes are the predominant mode of transmission whereas direct contact of animals with infected tissues (foetal or otherwise) may become predominant during the amplification stage of the epizootic (Pepin et al. 2010). In East Africa species which have been tested positive for RVFV using RT-PCR are *Aedes mcintoshi/circumluteolus, Aedes ochraceus, Aedes vexans, Aedes pembaensis, Aedes Pembaensis, Culex poicilipes, Culex bitaeniorhynchus; Culex quinquefasciatus, Culex univittatus, Cx. univittatus, Cx. Bitaeniorhynchus, Anopheles squamosus, Mansonia uniformis;* and *Mansonia africana* (Logan et al. 1991; Sang et al. 2010). Mosquito species identified to transmit RVFV in other parts of Africa include *Aedes juppi, Ae. caballus,* and *Ae. Linneatopennis* in South Africa (Métras et al. 2013), *Culex pipiens, Aedes vexans, Ae. Ochraceus* and *Ae. Dalzieli* in West Africa (Fontenille et al. 1998; Zeller et al. 1997). Even though the distribution of these vectors differ from one part of Africa to the other, they all use the same type of breeding sites and also feed on domestic ruminants.

The Rift Valley fever virus is thought to be maintained in nature at least in part by trans-ovarial transmission in flood water by Aedes mosquitoes during excess rainfall leading to floods referred to as '*dambos*’ (Dighe et al. 2010; Jost et al. 2010; LaBeaud et al. 2007). In turn, it results into an abundance of vector mosquito species (Breiman et al. 2008). Elfadil et al. (2006) showed a positive association between RVF outbreaks and a dense mosquito population, high rainfall and the presence of lakes and/or ponds. Between epidemic waves, RVF virus circulates at very low incidence without noticeable clinical manifestation, neither in human nor in animals (FAO EMPRES WATCH 2007). Rift Valley fever epidemics have been observed at irregular intervals of about 5–20 years (FAO EMPRES WATCH 2007; Ibrahim et al. 2008). Early entomological field investigations of the virus or increased activity of virus in the vector population is one of the key element in controlling RVF (Hall et al. 2012). In East Africa, RVF outbreaks are known to be closely associated with heavy rainfall events (Soti et al. 2012). Therefore, the prediction of RVF occurrence should be accompanied by satellite measurements of global and regional elevated sea surface temperatures, elevated rainfall, and satellite derived-normalized difference vegetation index data (Anyamba et al. 2010). Also the assumed importance of temporary ponds and rainfall temporal distribution especially during inter-epidemic periods needs to be investigated for effective control strategies of the vectors. Currently the inter-epidemic infection of RVFV in domestic animals is increasingly being reported in different parts of Africa which in most cases passes undetected (Heinrich et al. 2012; Sumaye et al. 2013).

The movement of domestic animals on the other hand has been reported to facilitate the transmission of RVFV from one place to the other during the epidemics and inter-epidemics. The appearance of RVF outside the African continent (Saud Arabia and Yemen in 2000) is said to be due to animal trade movements (Balkhy and Memish 2003; Ibrahim et al. 2008; Jansen van Vuren and Paweska 2009). Therefore, the movements of animals during epidemics should be restricted to prevent the spread of RVF to uninfected areas.

RVF causes storm abortions in pregnant animals and a high mortality approaching 100% in young animals (FAO 2000; Ikegami and Makino 2009). Sheep are more susceptible with more effect than in other ruminants (Elfadil et al. 2006). In humans, RVF causes a severe influenza-like illness characterized by fever (37.8–40°C), headache, muscular pain, vomiting and extreme weight loss (FAO 2000; Mohamed et al. 2010) with mortality rate less than 2% (LaBeaud et al. 2008). The effects of infections on human health are usually greatest on herdsmen and farm workers who live in close proximity to their animals, veterinarians, abattoir workers and butchers as an occupational hazard by direct handling of infected animals and their products (Isaäcson 2001).

In Tanzania the 2006–07 RVF outbreak was reported to the Arusha Veterinary Investigation Centre (VIC) on 21st January 2007 by the District Veterinary Officer (DVO) in Ngorongoro District who observed an abnormal disease in the district with massive abortions and deaths in animals suspecting to be RVF. Copies were sent to the Director of Veterinary Services (DVS) in Dar es Salaam, Regional administrative secretary (Arusha) and to the District Council Executive Director (Loliondo). This official report was followed by the local investigations done by the DVO. Reported cases from the livestock keepers in the district were after observing cases of abortions that started in December 2006 during rainy season. After epidemiological and clinical investigations by the veterinary district office, areas which had massive abortions and deaths in animals included; Pinyinyi, Monic, Engaresero, Matale A and B, and Malambo in Ngorongoro district. The first three villages lie along the floor of the Rift Valley along shores of Lake Natron (594–637 m above sea level) while Malambo and Matale A and B villages are on the escarpment of the Rift Valley. All the affected villages had heavy rainfall that started in December 2006. Engaresero village was also the first area to report RVF in 1998 outbreak. A team of experts from the Veterinary Investigation Centre (Arusha) were sent in the suspected areas in the district to carry out investigations and to collect specimens from suspected clinical cases of RVF. Specimens were dispatched to Onderstepoort Veterinary Institute, South Africa and at Tanzania Veterinary Laboratory Agency (Tanzania) where both laboratories confirmed RVF based on samples submitted. Apart from Arusha, there were other areas in the country that at the same time were reporting unusual abortion cases in sheep and goats. These areas included Manyara, Kilimanjaro, Tanga, Dodoma, Iringa and Morogoro regions reporting cases at different time intervals. The first two human RVF suspected cases were admitted on 28th January 2007 at Mount Meru hospital being from Terrat (Simanjiro district) and Makuyuni (Monduli district) in Manyara region. Sadly both of them died on 31st January 2007. Samples from these two patients were carefully collected and sent for detailed diagnosis to the Centres for Disease Control and Prevention (CDC) laboratory in Nairobi, Kenya and both were confirmed to be positive for RVF. Rift Valley fever was officially declared to the community in the country and OIE on February 7 and 12, 2007 respectively. In Tanzania, by the end of the outbreak in July 2007 it affected 10 of 21 regions of the country and 25 of 126 districts (Ibrahim et al. 2008; Swai and Schoonman 2009). There were 144 deaths of people out of 511 suspected cases (28.1% case fatality rate), whereby 186 (36.4%) were confirmed through laboratory tests and 124 (24%) classified as probable cases (Mohamed et al. 2010).

RVF remains to be a threat to livestock keepers and nations where the disease is occurring due to its major economic implications through the costs of the measures taken at individual, collective and international levels in order to prevent or control infection and disease outbreaks Otte (2004). However, there are few studies that have examined the socio-economic effects of the past outbreaks of RVFV, which reflects a lack of research focus on the broader social effects of the disease (Dar and McIntyre 2013). The socio-economic impacts, caused by morbidity and mortality of livestock and disruption of livelihoods, markets, and the meat industry that resulted into a ban of livestock slaughter and export of animals and animal products in Tanzania were not studied thoroughly during the 2006–07 outbreaks. Thus, the main aim of this study was to establish the socio-economic impact of RVF and assess knowledge, attitudes and practices to RVF control practices in selected areas of Tanzania using the 2006–07 outbreaks as a case study. Since natural outbreaks of RVF disease are sporadic, explosive with a very small window to allow effective planning and proper management of the disease during the outbreaks, the information obtained in this study will help the government to design preparedness programmes for effective control strategies for RVF disease. This in turn will impact positively on the livelihoods of livestock keepers who either depend on sales of live animals in the pastoral areas or those who keep dairy cattle and lose milk revenue whenever there is an outbreak of RVF.

## Results

### Socio-economic activities and benefits

In this study, 15 households reported to be purely pastoralists and 59 to be agro-pastoralists and their main sources of income being livestock keeping 73 (98.6%), agriculture 58 (78.4%), business 18 (24.3%) and employment 26 (35.1%). The categories of livestock kept by majority of households in the area are cattle, goats, sheep and chickens and those kept by minority are donkeys, pigs, dogs and cats (Table 1). Pastoralists were found to keep cattle, goats, sheep and donkeys, while animals kept by agro-pastoralists in additional to those kept by pastoralists included chickens, pigs, dogs and cats. In this study 67.6% of the respondents reported to have inherited animals from their livestock keeping families.

**Table 1:** **The total number of different animal categories and the average number kept per household in the study households**

Animal category	Total number of animals	Average number kept per household	
		Pastoralist	Agro-pastoralist
Cattle	6 234	64	20
Goats	4967	52	15
Sheep	3997	43	11
Donkeys	31	0.8	0.04
Chickens	225	0	9
Dogs	18	0	0.72
Cats	8	0	0.32
Pigs	186	0	5

Local breeds (Table 2) were kept by the majority livestock household keepers. Cattle were the domestic animals that made by far the greatest contribution to livestock-based livelihoods in the study area. In the case of agriculture, crops that were cultivated included maize, beans, banana, potatoes, rice, finger-millet, sorghum, green gram, sunflower, pigeon peas, cow peas, chick peas, cassava, onions, and vegetables. Among the mentioned sources of income, livestock keeping gave them more income 53 (71.6%) followed by agriculture 12 (16.2%), while 9 (12.2%) thought that both livestock keeping and agriculture had equal contribution to income generated.

**Table 2:** **Type of animals kept in the study area (N = 74)**

Animal category	Animals kept (%)	Animals kept by majority (%)
cattle	94.6	93.2
goats	90.5	93.2
sheep	78.4	78.4
chickens	37.8	25.7
donkeys	41.9	38.5
dogs	27.0	6.80
cats	16.2	2.70
pigs	5.40	1.40

**Figure 1:**
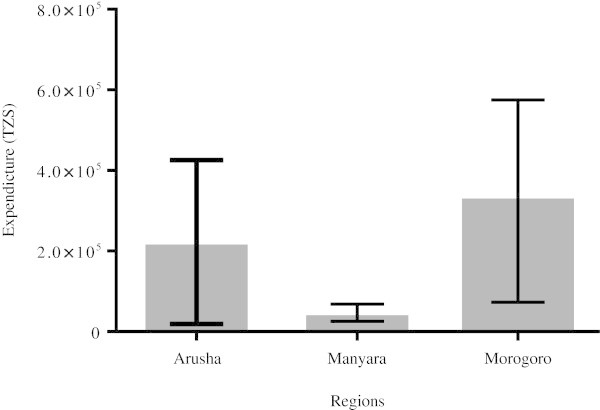
**Representation of an average monthly expenditure of livestock households in the study area (US $1 to 1500 TZS).** Expenditure levels were not significantly different between the regions (P = 0.414) and within the regions (Arusha P = 0.0564, Manyara P = 0.0668 and Morogoro P = 0.3522).

The minimum, average and maximum expenditure per month of livestock households in the study area were found to be 25 000, 120 000 and 3 000 000 Tanzanian shillings (TZS) respectively at the rate of US$ 1 to 1500 TZS. The highest expenditure was observed in Morogoro (from a pastoralist) and the lowest being in Manyara region from an agro-pastoralist (Figure 1).

The main benefits derived from livestock keeping were reported to be food (meat, milk, ghee, and fat), socio-cultural roles such as paying dowry, school fees and buying school needs, draft power, buying household requirements and health (Table 3). Other benefits included transport for donkeys, skins, manure for crop production and building houses. Thirty (40.5%) livestock keepers used manure for crop production and the majority left it in the yard (47, 63.5%); few disposed off (11, 14.9%), sold to others (5, 6.8%) or use for decorating their houses (10, 13.5%). The amount of manure used for crop production ranged from 100 kg to 20 tonnes annually. Draught cattle were used for cultivation and or tracking luggage for an average of 3 to 5 hours per day. The average amount of milk obtained per household per day was 19.4 litres; the amount differed from one household to another depending on the number of animals kept. Only 36 (48.6%) sold their milk for a price ranging from 300 to 2000 TZS (US$ 1 to 1500 TZS). Livestock keepers earned most of their income from selling animals. The price before, during and after RVF outbreak of 2006–07 varied significantly depending on the animal species (Table 4).

**Table 3:** **The purpose of keeping livestock as reported by livestock keepers in Arusha (n = 36), Manyara (n = 16) and Morogoro (n = 22)**

Advantage	Arusha (%)	Manyara (%)	Morogoro (%)
Paying dowry	81	44	64
School needs	97	94	95
Food	100	94	100
Agriculture	67	56	5
HHR*	100	88	100
Health care	97	81	100
Transport	25	13	5
Get manure	8	25	49

*HHR = Household requirements.

**Table 4:** **Average price of selling animals before, during and after RVF outbreak of 2006-07**

Animal category	Before RVF	During RVF	After RVF	P-Value	Comment
Bulls and Oxen	507 373	398 571	611 864	1.28 × 10^-5^	Significant***
Cows	328 276	267 059	40 4068	6.77 × 10^-4^	Significant**
Heifers	196 316	156 061	25 1754	1.44 × 10^-6^	Significant****
Calves	125 714	136 935	18 6786	0.0337	Significant*
Goats and sheep	40 918	36 135	61 475	1.86 × 10^-9^	Significant*****

*Indicates increasing strength of significant different in price of selling animals as the number of star increases where P > 0.05 considered significant.

### Livelihood constraints

The main constraints in the area were animal diseases (Table 5), drought, inadequate pasture, water availability and lack of dipping tanks. Two (22.2%) villages during focus group discussions reported to use dipping tanks and six (66.7%) to use spray pumps to control vector borne diseases. As reported by the FGD, inadequate water and pasture in 2009 caused high mortalities in animals. Consequently, pastoralists were forced to become agro-pastoralists in northern part of Tanzania in order to cope with the losses.

**Table 5:** **Proportion (%) of common and outbreak diseases reported in the study area by individual household (N = 74)**

Disease	Diseases encountered	Diseases with great loss	Diseases in outbreak form
East Coast fever	79.7	47.3	12.2
Malignant catarrhal fever	12.2	4.1	1.4
Trypanosomosis	50.0	6.8	1.4
Contagious Bovine Pleuropneumonia	45.9	24.3	13.5
Contagious Caprine Pleuropneumonia	60.8	40.5	9.5
Peste des Petits Ruminants	13.5	12.2	0.0
Rift Valley fever	2.7	0.0	36.5
Fasciolosis	10.8	4.3	0.0
Helminthosis	21.6	4.3	0.0
Anaplasmosis	17.6	6.8	1.4
Babesiosis	9.5	2.7	0.0
Anthrax	23.0	5.4	24.3
Myiasis	40.5	21.6	2.7
Foot and Mouth disease	32.4	2.7	6.8
Lumpy Skin disease	20.3	1.4	0.0
Heartwater	2.7	0.0	1.4
Black quarter	5.4	1.4	6.8
Brucellosis	0.0	0.0	1.4
Swine flue	0.0	0.0	1.4

### Community based knowledge on handling and control practices of RVF

The government used the community meetings to educate people on the presence of RVF disease in the country, how people get the disease and preventing them from eating uninspected meat. All people involved in slaughtering animals or handling slaughtered meat and livestock products were told to take all the necessary precautions. Great emphasis was given to livestock keepers to send their animals for vaccination. The community in the study area in addition received information on managing common and new diseases in the area from radio, few from LFOs and local government authorities. During the study, 69 (93.2%) reported to have heard about RVF in their life time and only 27 (36.5%) knew that it was an outbreak disease. Also 26 (35.1%) of the respondents reported that RVF disease outbreak happened in the study area and only 22 (29.7%) indicating the exact year of the last outbreak of 2006–07 with few (3, 4.1%) reporting the 1997–98 and majority failed to remember the year of the last outbreak. When asked on how RVF manifest in animals, some were able to mention the following signs; storm abortions, high fever, high mortality in lambs and kids, ocular and nasal discharge, haemorrhagic diarrhoea, vomiting, abdominal pain, jaundice and body swelling (Table 6).

**Table 6:** **The knowledge of livestock keepers on clinical signs of RVF in livestocks in Arusha (n = 36), Manyara (n = 16) and Morogoro (n = 22)**

Clinical sign	Arusha (%)	Manyara (%)	Morogoro (%)
Storm abortions	44	19	27
High fever	28	6	0
High mortality	36	6	14
Ocular and nasal discharges	39	13	23
Haemorrhagic diarrhoea	19	0	18
Vomiting	3	0	0
Abdominal pain	6	0	0
Jaundice	25	13	14

Respondents who reported to have the disease in their household were the one who could remember significantly the clinical signs of the disease (P = 0.002) and especially storm abortions, high fever, high mortality in young animals, and oculonasal discharges. On the side of the animals affected by RVF that were significantly identified in the household were goats (P = 0.001) and sheep (P = 0.002) probably because are the ones that were severely affected. Those who heard the disease from either neighbours, mass media, local government authority or livestock experts, most of them did not remember the clinical signs of the disease. This study has indicated that there was little knowledge on clinical signs of RVF and the difference in the three regions was statistically significant (P = 0.00459).

The presence of mosquitoes in villages lying on the shores of Rift Valley especially in the evening together with floods was associated with the outbreak of the disease. Cattle, sheep, goats and human being were reported to be affected by RVF and man could get the disease from eating meat and drinking milk of RVF sick animal. Many respondents could not remember the exact year of the 2007 RVF outbreak. Livestock keepers who experienced the disease treated animals themselves using oxytetracycline, but there was no response. Respondents reported that animals were not vaccinated against RVF before the outbreak of 2006–07 as the government did not have such a control programme in their area. Livestock keepers understood that vaccination was important for controlling livestock diseases and most of them were ready to vaccinate and fully participate in the programme. During the outbreak, vaccinations were done in areas where there was no disease and targeted goats and sheep which were severely affected.

### Socio-economic impact of RVF

The disease posed a great threat not only to the livestock keepers but also to the Government due to its social and economic implications. There were costs incurred due to measures taken at different levels in order to prevent or control infection and disease outbreaks. Rift Valley fever affected people in the study area two-fold; directly and indirectly. Directly, livestock keepers lost their animals through deaths (Tables 7 and 8) and massive abortions (Table 8), and lost all the benefits mentioned previously (Table 3).

**Table 7:** **Status of livestock in the study households during RVF disease outbreak in the three regions**

Animal category	Total No	Diseased animals	Treated animals	Animals died	Vaccinated animals
Bulls	594	37	21	21	302
Oxen	145	0	0	0	85
Cows	2398	38	38	20	1383
Heifers	403	21	21	13	248
Calves	1127	249	236	121	9
Goats	2721	128	108	50	1759
Kids	1276	205	199	164	0
Sheep	2516	49	49	35	0
Lambs	1191	133	123	119	0

**Table 8:** **Overall deaths and abortions in domestic ruminants during the 2006/07 RVF outbreak in the study districts**

			Deaths N (%)			Abortions (Total)	
Region	District	Cattle	Goat	Sheep	Cattle	Goat	Sheep
Arusha	Ngorongoro	424780 (0.29)	437103 (0.34)	327424 (0.39)	1439	1757	1314
	Longido	302272 (0.27)	391953 (0.26)	305797 (0.32)	1027	1254	1024
	Monduli	283428 (0.21)	368223 (0.20)	294395 (0.28)	776	948	847
	Arumeru	128355 (0.11)	318095 (0.05)	240915 (0.04)	143	175	101
Manyara	Simanjiro	482810 (0.10)	295883 (0.20)	148064 (0.23)	786	960	554
Morogoro	Mvomero	132560 (1.10)	98245 (1.83)	19797 (3.23)	1219	1488	559
	Kilosa	156246 (1.48)	122609 (2.33)	38542 (3.25)	1166	1423	522

Source: District veterinary offices and Veterinary Investigation Centre (Arusha). N = Total number of animals in a district, the numbers in brackets were the percent of deaths.

Non-livestock keepers were affected directly from lack of red meat as most of the markets were closed (45, 60.8%) in many areas of the country and also they were also affected by fear stress as the disease was politically exaggerated. Also indirectly non-livestock keepers were affected by competing in other sources of food which replaced the red meat such as chicken, sardines, pork, vegetables, fish and beans. The price for these replacements became high making low income people failing to buy them creating another big problem of malnutrition due to lack of protein-rich food. However, none of the respondents reported a household which stopped keeping animals because of the impact caused by RVF in the study area. Turning to agro-pastoralist was their copying strategy after the outbreak.

Reports from Tanzania Veterinary Laboratory Agency (TVLA) indicated that there were deaths of 16 973(0.10%) cattle, 20 913(0.18%) goats and 12 124(0.31%) sheep and 15 726(0.09%) abortions in cattle, 19 199(0.16%) in goats and 11 085(0.28%) in sheep. Both reports from District veterinary offices and Arusha VIC and TVLA show that sheep were highly affected followed by goats then cattle. Livestock keepers highly depends on their animals for their daily needs (Table 3), they sell their animals so that they can sustain their needs. The changes that took place during the disease in terms of price for selling animals greatly affected livestock keepers. During the RVF outbreak the average price for the different category of animals went down and became higher after the outbreak except in calves where the price increased progressively (Table 4).

### Psychological distress of diseased families

The livestock household that were affected by RVF in 2006–07 faced psychological distress associated with the disease. The psychological distress included loss of living confidence (fear of death), possibility of contracting the disease, possibility of losing animals as they depended on them, fear of eating meat and loss of livestock market due to legal restriction of livestock markets in and outside the country. During focus group discussion, it was reported that, not only the diseased families who appeared to experience psychological problems, but many individuals which included livestock and non-livestock keepers. Psychological distress was reported during questionnaire survey to be more severe in families that had RVF in their households. In this study, the households whose livestock had RVF were 18 (50%), 6 (38%) and 10 (45%) in Arusha, Manyara and Morogoro respectively.

### Challenges for controlling RVF outbreaks

This study has revealed some challenges for managing animal and human disease disasters in the country. These challenges need to be addressed for effective future control RVF outbreaks. The main challenges that have been observed in this study are as follows:

### Housing

**Figure 2:**
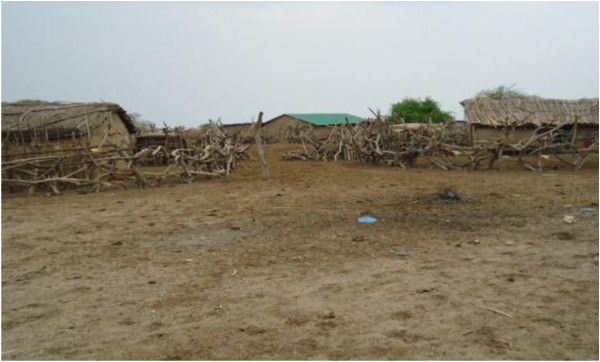
**Representation of Maasai community village (Monic village, Ngorongoro district in Tanzania) on the Eastern arm of Rift Valley shared houses with domestic animals during the outbreak.**

Most human and animal housing in the pastoral and agro-pastoral systems in Tanzania are not reliable and more so for animals. As it was observed during the study (Figure 2), some communities had their houses at the centre of the animal’s house while others were just close to the human houses and open. This was reported during in-depth interview with the key informants as one of the factors that contributed to the occurrence of the first RVF human cases in the pastoral settings as in the intensive farming systems animal houses were in good condition and well closed.

### Inadequate knowledge on control methods for RVF

This study has indicated that only 2.7% (2) of the respondents got knowledge about RVF and that 10 (13.5%) knew that there was a control measure currently in place that involved vaccination. The level of literacy in the study community was low due to nomadic lifestyle with 37.8% (28) being illiterate, 39.2% (29) standard seven, 5.4% (4) form four, and 1.4% (1) of the respondents being college graduates. This has an impact not only in the transmission and implementation of control strategies for RVF but also in the control of other livestock diseases.

### Control of animal movements

Animal movements contribute very much on the spread of RVF from one village to another. During the 2006–07 outbreaks in Tanzania, animal movements were restricted and local government authorities reinforced regulation about animal movements. However, there were some people who moved animals from one village to another in search for pastures and water with few for search of livestock markets in near village, district, region or country (Kenya). Only five (6.8%) respondents reported to control animal movement in their households during the outbreak and they were agro-pastoralists.

### Treatment of animals by livestock keepers

Veterinary services in agro-pastoral and pastoral communities in Tanzania are mainly provided by LFOs who are found at least in each ward. However, provision of service to animals has been very minimal due to uncontrolled animal movements and treatment by farmers. During the outbreak of RVF in 2006–07, 17 (23%) of the households treated their animals either themselves (14, 18.9%) or LFOs (2, 2.7%). During focus group discussions, farmers said that they treated animals because it was expensive to call LFOs who demanded payments for fuel and drugs. The knowledge for treating animals by themselves was acquired from the family members and other livestock keepers. Free market economy for veterinary drugs led to easy access. Some veterinary drugs are sold in open markets ('*minada*’) sometimes in direct sun rays. These drugs came from local veterinary shops available in Tanzania and some from Kenya (farmers from the northern part). This has great impact in the control of diseases especially outbreaks as they will report after so many trials while the disease is spreading to other animals and households or villages. The main drug for treatment for many diseases in the households was Oxytetracycline (OTC). This drug was used to treat RVF cases but there was no response at all.

### Consumption habits of meat and milk

In pastoral and agro-pastoral communities meat inspection is not commonly practiced hence respondents reported consumption of meat without inspection as a normal practice. Both confirmed human cases in Arusha and Manyara as reported by key informants and focus groups was due to consumption of meat from dead sheep. Despite the local government authorities prohibiting people from eating meat without inspection and drinking unpasteurized milk during the outbreak, some people continued to eat meat and drink raw milk.

### Insufficiency of dipping tanks

The use of acaricides to control ticks and other ectoparasites in the pastoral and agro-pastoral farming systems is not effective due to their nomadic life styles. Focus groups indicated that only two villages (N = 9) had dipping tanks and the remaining villages controlled ticks by spraying animals. The method of controlling ticks by spraying animals is tiresome especially for a large group of animals.

### Delay on emergency plans for controlling RVF

There was a complaint from some key informants that they were not involved during initial stages of preparations of emergency plans despite the fact that they knew the areas of outbreaks very well. As being experts in the areas, they could advise the government appropriately regarding important areas for vaccinations, because some vaccinations were done in areas where it was not necessary or of priority. There was a delayed response following communication with government officials. This was attributed to either long chain of command or slow acting of the responsible people (administrators) along the chain or not having emergency plan for RVF. Early warning message were issued by EMPRES in November 2006 predicting RVF outbreak in sub-Saharan Africa based on predictive climatic models like NDVI (Normal differential vegetation index), elevated temperatures in the Pacific and Indian oceans that indicated heavy rains, elevated humidity, and cloud cover favouring increased population of mosquitoes that support and spread RVF virus (FAO EMPRES WATCH 2006; Martin et al. 2007; Dijkman et al. 2009). This information was not acted upon on time in Tanzania. The disease outbreak started in late November 2006 while vaccinations started early march 2007. The human resource was available but the problem was a delay in accessing funds, equipment, and vaccines. During the outbreak, vaccines came very late and were insufficient to cover the number in infected areas. In some occasions vaccines came but there were no funds to allow vaccination campaigns to start.

### Lack of coordination and inter-sectorial collaboration

During in-depth interviews the key informants reported that the control of RVF was highly influenced by politicians. Vaccinations were carried out in areas which did not qualify based on vaccination regimes. In this case areas with infections could be vaccinated boosting up the disease. There was no clear mode of coordination between the central government (Ministry), and local governments (Districts) on inputs distributions. Some inputs were distributed directly to districts, some to veterinary investigation centres with no or little harmonization and coordination. The link between livestock sector and public health sector was inadequate especially on disease diagnosis and control.

### Low diagnostic capacity

During the outbreak, samples from human being and animals were taken to Kenya and South Africa for confirmation. This caused a delay on official declaration on the presence of disease and containing the disease in the country leading to more socio-economic effects. By the time of last outbreak the country did not have a level three biosafety laboratory where tests for RVFV could be handled. Diagnosis of RVF in the country depended on clinical signs and ELISA tests at TVLA in Dar es Salaam.

## Discussion

Findings from this study have revealed that the majority of farmers think livestock keeping give more income than cropping. Farmers in the study area depend on livestock as the main source of income. However, diseases and drought pose serious threats to livestock keepers. Losses are attributable to morbidity, mortality and costs of disease treatment and control measures to meet national and international requirements. Epidemic diseases such as RVF, with few natural factors to limit their spread and experience in managing them bring great threat to livestock keepers. Tanzania has an estimated livestock population of 17 million cattle, 11 million goats, and 3.6 million sheep (Mohamed et al. 2010) most of which are located in the north and central regions of the country. These regions were severely affected by the 2006–07 RVF outbreak leading to disrupted socio-economic setting of all Tanzanians and more so livestock keepers who are completely dependent on livestock and their products.

The 2006–07 RVF outbreak in Tanzania started in the northern part with abnormal abortions and deaths in domestic animals in late December 2006 and confirmed in January 2007. In early February 2007, the Government of Tanzania held an emergency inter-ministerial meeting in Arusha after which the District Commissioners were given tasks to prepare strategies to control the disease in their districts. One of the strategies was to provide education to the community on clinical appearance of the disease, spread of disease (transmission) and the effect of disease to human and their animals. Also education was given in slaughter premises to all people who were involved in handling and slaughtering animals. Livestock keepers were emphasized to make sure they did not move animals from one village to another and that they were to participate fully in vaccination campaigns.

During this time the government was ordering Smithburn vaccines abroad and organizing funds and human resources. The first vaccine doses were received by the government in the end of February 2007, 116 600 were distributed to districts with reported cases namely Monduli, Ngorongoro, Simanjiro, Longido, Hai, Babati, Mkinga and Kilosa. Also equipments and funds to run vaccination campaigns were provided by the Government. Additional, 370 400 vaccine doses were distributed to all districts as well as in other two districts namely Iringa rural and Mvomero in which RVF cases were reported. Vaccination campaigns to animals started early on March 2007 to all ages of cattle, sheep and goats except those under six months and pregnant animals were vaccinated. Emphasis was put on sheep and goats when the amount of vaccine was not enough. Vaccinations started on the high risk areas for RVF and ended on the low risk areas based on the known history of RVF outbreaks.

This study has revealed that, during the outbreak minimal education was given to the community in the study area and more so to the pastoralists who live nomadic life. Pastoralists depended much on radios to get information about RVF as they can carry with them even to the remote grazing areas. The government used community meetings, posters, newspapers and seminars to educate communities. Since during this study, most of community members were found with low knowledge about RVF may imply that, the education provided was not effective. It has been reported by other researchers in Tanzania and elsewhere (Cripps 2000; Fyumagwa et al. 2011; Swai et al. 2010) that, it is not only livestock keepers, but also veterinary field staff and staff in health facilities, have a low awareness and poor knowledge of zoonoses. In livestock keeping community, the majority of them had not gone to school and therefore posters were not suitable for them. In this regard, providing education via their local leaders and radios could be the best option for livestock keeping community to get education easily during RVF outbreak. Vernacular languages should be used when providing education and information to the pastoralist as it has been seen that there was good proportion of people who could not understand and speak the national language (Kiswahili). This observation was also noted in Kenya (Munyua et al. 2010) as among obstacles for efficient dissemination of information and extension of knowledge to livestock keepers. With advancement of communication technology, the use of automated messages via mobile phones that would provide information on outbreak of diseases may be useful. Majority of the livestock keepers nowadays in Tanzania own and use mobile phones for their family matters and seeking market information. The application of mobile technologies by the livestock keepers and veterinary professionals to exchange information on livestock diseases will enhance disease surveillance (Karimuribo et al. 2011a).

During the 2006–07 RVF outbreaks, some farmers went on eating meat without inspection and proper cooking that led to more human cases especially in some parts of Dodoma. This was due to their socio-cultural behaviour of eating meat not inspected or from dead animals. This calls further educational intervention at community levels. The limited knowledge of pastoral communities on risk practices including eating raw meat, raw milk, touching and herding aborted animals and consuming products from animals predisposes them to zoonotic diseases (Anyangu et al. 2010). On the other hand in some communities it was observed that human and animals shared the same housing that also predisposed them to zoonotic diseases. This has also been observed and associated as one of the risk factor for transmitting zoonotic diseases (including RVF) to human (Jost et al. 2010; Swai et al. 2010). Hence more education is needed and interventions that will enable the community live in separate houses from animals.

In pastoral communities, animals that get sick are often treated by themselves due to unavailability of livestock disease professional and para-professionals that can take charge in disease diagnosis, treatment and other disease management. Other factors include high treatment cost linked to calls of veterinary doctors and buying of drugs, keeping large number of animals just for prestige, nomadic lifestyle and insufficient knowledge on best ways to control diseases. Apart from livestock experts being few, nomadic lifestyle contributes by far for the limited access to veterinary services that would provide service on time. Pastoralists have limited knowledge about dosage and routes for drug administration. Free market economy for veterinary pharmaceutical in Tanzania contributes greatly to self-treatment of animals by and mishandling of drugs. Easy access to drugs and self-treatment procedures have great impact on control of livestock diseases especially during outbreaks as pastoralists will report after so many trials, while the disease is progressing to spread. The use of trained Community Animal Health Workers (CAHWs) as an important alternative animal to animal health delivery channel in the country’s marginal areas where there are few professional veterinary practitioners will help to reduce the problems (Allport et al. 2005; Swai and Masaaza 2012). In the rural settings health delivery systems are hampered by many factors including remoteness, poor infrastructure, inadequate transport, lack of qualified veterinary staffs and insufficient funds to support surveillance operations and buy reagents and drugs (Swai and Schoonman 2012). Therefore, the use of CAHWs could be a good link to the veterinary professionals and the livestock disease control units for providing information to the livestock keepers and to the veterinary experts.

During the outbreak, the quarantine was not executed properly as pastoralists could still move their animals from one village to another to search for pastures. Also farmers were still selling animals to nearby country (Kenya) via unauthorized routes. The movement of animals from Tanzania to Kenya either for search of good pasture or for sale has been reported (Diallo et al. 2000) to facilitate further spread of the disease to unaffected areas during the 2006–07 RVF outbreak. It was observed that good pastures were found in the low land areas where mosquitoes were also found in large numbers and facilitated the disease transmission. However, the short difference in time of occurrence of disease in different regions of Tanzania is an indication that, those foci of outbreaks were caused by other factors other than animal movement as it was also highlighted by (FAO 2000). The results also indicated that few farmers used dipping tanks and the majority used spray pumps to control vector borne diseases. Since some of the farmers owned large number of animals, it was not possible to effectively spray all of them. According to (Peter et al. 2005) and (Davies and Martin 2006) effective use of dipping tanks also reduces the magnitude of mosquito borne diseases like RFV.

Rift Valley fever led to disruption of whole market chains system in the country similar to what it was reported in other countries that experienced the disease (Holleman 2002). The study has indicated that sheep were highly affected followed by goats then cattle as it was observed in Kenya (Jost et al. 2010) following similar outbreaks. This was contributed by lack of emergency plans that led to delayed control of RVF in the country. This was a similar observation in Kenya (FAO EMPRES WATCH 2006; Martin et al. 2007). Thus, there is a need of having organizational rearrangement so that an emergency unit is put in place that will deal with emergency diseases especially outbreaks or unknown cases that require fast action to prevent massive socio-economic loses. The normal administrative structures of national veterinary services that deals with routines animal health programmes have been reported (FAO 2000) to be ineffective for emergency cases. During the 2006–07 RVF outbreak in Tanzania, the coordination for controlling the disease was under the umbrella of the National Disaster Preparedness and Response unit within the Prime Minister’s Office (Karimuribo et al. 2011b). Establishment of the unit will ensure active surveillance and monitoring is carried out routinely in the field to create baseline information on inter-epidemic virus transmission patterns, areas at risk and early warning of RVFV activity or increased mosquito populations. Also annual vaccinations in highly susceptible areas identified by experienced livestock stakeholders are done together with early distribution of enough vaccine doses, equipments and funds during outbreaks.

Control measures that were put in place by the government could not be implemented properly because time frame for the disease to spread in a wider area was very short to enable the government to provide education as fast as possible. Inadequate numbers of livestock disease experts in the livestock keeping community led to livestock keepers treat their animals. This led to delayed reporting of the disease outbreaks. Also lack of collaboration between and within the livestock sectors led to difficulties in effective implementing control measures during the outbreak. Thus, there is high chance that the disease disappeared naturally. Since RVF affects human, domestic and wild animals and transmitted by arthropods, the approach towards its control should involve a number of government Ministries. In Tanzania the Ministries includes, the Ministry of Livestock Development and Fisheries, the Ministry of Health and Social Welfare and the Ministry of Natural Resources and Tourism. During the 2006/07 RVF outbreak, the Ministries responsible for Livestock Development and worked in isolation and in ad hoc manner using Ministerial contingency plans which also have no common point of intersection (Fyumagwa et al. 2011; Mbugi et al. 2012). There is a need of creating a point of intersection in order to be able to fit in the concept of 'One Health Approach’ which is thought to be a better way of combating infectious diseases. The initiatives towards the One Health infectious diseases surveillance in Tanzania has been started by introducing Masters in One Health Molecular Biology at Sokoine University of Agriculture under the Southern African Centre for Infectious Diseases Surveillance (SACIDS). The Government of Tanzania launched officially the One Health Approach in Arusha on April 16, 2013 by the Vice President of the United Republic of Tanzania.

## Conclusion

Rift Valley fever is a multi-disciplinary disease which demands a one heath approach in order to control it during the outbreaks. Much is needed and can be done by education, and in particular by increasing the awareness of different health professionals, and facilitating communication and collaboration between veterinary, public health and agricultural personnel on RVF. To achieve this, there must be a point of intersection in the Ministerial policies, Acts and Contingency plans that tries to address how to deal with zoonotic diseases. There is a need of establishing active surveillance system for RVFV which will capture the increase of virus activity in the vectors together with the use of other weather monitoring station (the forecast models) like the NASA Meteorological information. Annual vaccinations of domestic animals especially in areas known to be the hotspots of RVF outbreaks should be done. These should not wait for the outbreak to occur because there is very small window to prepare before the disease spreads to a wider area leading to tremendous effects.

## Materials and methods

### Study area

**Figure 3:**
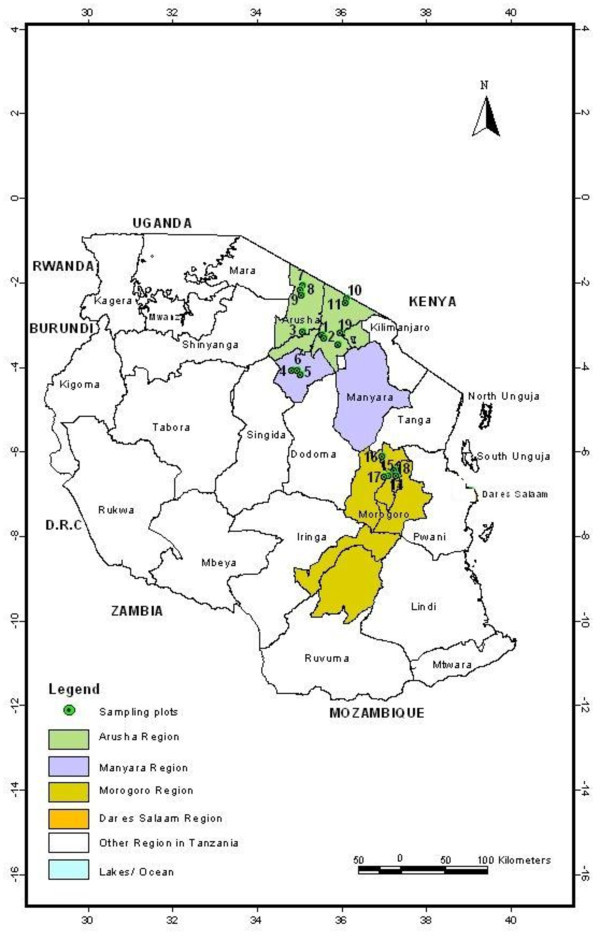
**Representation of the map of Tanzania showing study areas: number 1 to 19 shows locations where socio-economic study was conducted in Arusha, Manyara and Morogoro regions.**

The study was carried out in Arusha and Manyara where pastoral farming is practiced and Morogoro where dairy and agro pastoral farming systems are practiced in Tanzania (Figure 3). Arusha, Manyaara and Morogoro have an altitude ranging from 482 to1368 m above sea level and are among the areas that experienced RVF outbreaks in 2006–07. These areas normally experience two rainy seasons: a short rainy season between October and December, and a long rainy season between March and May. Typically, the annual precipitation averages between 500 and 1000 mm. The vegetation consists mainly of various shrubs and acacia bushes, and livestock species kept are primarily cattle, goats and sheep.

### Research design

A cross-sectional study design that allows data to be collected at a single point in time was used in this study to collect data between January and April, 2012. The following formula for sample size estimation as proposed by Naing and his colleagues (2006) was used for socio-economic study;1

Where n = estimated sample size, Z = Z statistic for 95% confidence interval (1.96), P = expected proportion of livestock household keepers with knowledge on RVF (13%) based on findings by (Labeaud et al. 2008) and d = is the margin of error set at 95% (5%). This gave a total number of 174 livestock household keepers.

### Sampling technique

In this study a multi stage sampling method was employed. A purposive sampling method was used to obtain regions based on pastoral (Arusha and Manyara regions), dairy and agro pastoral (Morogoro region) farming systems. Also these are areas which were affected by the 2006/07 RVF outbreak. The villages (19) included in this study were drawn randomly using random number generator built in Microsoft Excel 2010 from a frame of all villages in the three regions. Individual households within the villages were selected based on keeping livestock and having kept animals for more than ten years. Before commencement of the study, the questionnaires were pre tested to check the validity and how the individuals could understand and respond to questions. The questionnaires were modified on the basis of the result of the pre-test. The pre-test used a total of ten (10) livestock households in one village (Sokoine in Mvomero district) with five (5) households being pastoralists and five (5) households being agro pastoralists.

### Data collection

During the study, quantitative data were collected using questionnaire while qualitative data were collected using in-depth interviews with key informants and focus group discussions. The questionnaire was pre-tested in six farms, three for each pastoralists and agro-pastoralists.

#### Quantitative data

The questionnaire survey used 74 respondents with age ranging from 21–79 years old. The participants were interviewed on socio-economic and cultural activities, type of livestock kept and livestock involvements regarding RVF disease impact on household livelihood activities. The seasonality of both human and livestock RVF issues, trade and marketing in livestock, their product and their perception on the occurrence of RVF on their livelihoods were assessed. The study determined the social organization of production; livelihood constraints; household’s income sources and average monthly expenditure; number of livestock-holding households and stock of livestock in the households including data on number, species and breeds as well as the quantity of livestock products (milk, meat, manure, traction power) produced and marketed by livestock keeping households through the year. Furthermore the community based knowledge for management of RVF was established to explore on what the community know and what was implemented during the 2006–07 outbreak. The interviews also collected descriptions of the clinical presentation of RVF in people and livestock, as well as its incidence relative to other diseases.

#### Qualitative data

These were general information about livestock diseases (disease outbreaks, specific view on RVF disease, control, and its significance to livestock, and government involvement to control of RVF). Also issues on livestock regulations and reasons for success and failure to implement recommended management procedures were explored during in-depth interviews. In-depth interviews were conducted with district veterinarians, veterinary investigation centre officers and LFOs who had been involved in the management of the 2006–07 outbreaks. The study also used focus group discussions (FGD) with agro-pastoralists and pastoralists in some villages where questionnaire was administered. The focus groups involved between 5 to 12 people, most of whom were men and most were ethnically Maasai with few Mbulu, Barbaig and other tribes. Nine focus groups (three from each region) were conducted. The groups were introduced to the research topic before starting the discussion and the duration of discussion was between 30 and 60 minutes. The discussion was guided by a set of prepared questions and the permission to document and record the discussion was obtained from the participants. The focal group participants were interviewed on their economic and cultural activities, knowledge on the impact of livestock diseases and their management, responsibility for disease control, awareness on outbreak diseases especially RVF and how the community obtain general information about outbreak of diseases.

#### Data from government offices

Information on the areas affected by RVF, total number of animals died and aborted, emergency plans and the stake holders involved during the outbreak were obtained from district and regional veterinary offices, Arusha Veterinary Investigation Centre, Tanzania Veterinary Laboratory Agency and the Ministry of Livestock Development and Fisheries.

### Data analysis

In this study, Statistical Package for Social Science (SPSS) version 17.0 was used for descriptive analysis (means, frequencies) and comparing the proportions for data collected using questionnaire. Analysis of variance (ANOVA) was used to compare means between populations. The MAXQDA 10 was used for analysis of focus group discussion transcripts.
